# A novel augmentation technique for the repair of full thickness gluteal tendon tears: a biomechanical analysis in an ovine model

**DOI:** 10.1186/s10195-025-00850-1

**Published:** 2025-05-24

**Authors:** Alexander Derksen, Zarife Balli, Henning Windhagen, Dennis Nebel, Janin Reifenrath

**Affiliations:** 1 Department of Orthopaedic Surgery, Hannover Medical School, DIAKOVERE Annastift, Anna-Von-Borries-Str. 1-7, 30625 Hannover, Germany; 2 Laboratory for Biomechanics and Biomaterials, Department of Orthopaedic Surgery, Hannover Medical School, DIAKOVERE Annastift, Anna-Von-Borries-Str. 1-7, 30625 Hannover, Germany; 3 Lower Saxony Center for Biomedical Engineering, Implant Research and Development (NIFE), Stadtfelddamm 34, 30625 Hannover, Germany

**Keywords:** Gluteal tendon repair, Gluteus medius, Gluteus minimus, Augmentation, Tendon refixation, GTPS, Ovine model

## Abstract

**Background:**

Gluteus medius tendon tears lead to considerable functional limitations and a high level of suffering in affected patients. In cases where the symptoms are severe, surgical intervention is indicated. A range of techniques are used to repair the tendon, with the primary aim being to achieve the highest possible primary stability in order to minimise the risk of re-rupture. This biomechanical study compares two different refixation techniques in terms of their stability in an ovine model.

**Material and methods:**

The gluteal tendons of sheep hips (*n* = 17) were meticulously prepared and detached from the femoral insertion. To reattach these tendons at their original anatomical footprint, either the sole double-row transosseous-equivalent technique (DR) or the DR supplemented by a proximal suture insertion (augmentation) of the tendon (DR +) was used. Pull-out tests were performed until failure using a uniaxial material testing machine, with a tensile force applied along the physiological tensile direction of the hip abductors. The data obtained (force at failure, linear stiffness) were compared between the groups using the Mann–Whitney *U* test.

**Results:**

The augmentation of the proximal tendon portion resulted in a substantial increase in force at failure, exceeding 450% (698 ± 80.3 N DR + compared with 155.9 ± 53.9 N DR technique). In addition, augmented tendons exhibited a notable enhancement in stiffness, with an average increase of 31.3 ± 15 N/mm in DR + compared with 12.4 ± 4.8 N/mm in DR. Furthermore, the DR + method resulted in a substantial reduction in the incidence of slippage of the tendon fibres out of the sutures and tendon bundles when compared with the DR suture.

**Conclusions:**

The clinical problem of suture knots becoming loose within the tendon stump, leading to the failure of the tendon sutures, could be mitigated by additional augmentation, resulting in a substantial increase in ultimate load at failure. The benefits of the double-row transosseous-equivalent technique, which facilitates the pressing of the tendon stump against the footprint, are maintained.

*Level of Evidence* Level of Evidence 5

**Supplementary Information:**

The online version contains supplementary material available at 10.1186/s10195-025-00850-1.

## Introduction

Gluteus medius and minimus tendon tears can result in peritrochanteric pain and functional limitations in the hip joint [1]. The limitations include a reduction in abduction ability and diminished pelvic stability during the stance phase. The aetiology of these limitations is multifactorial, ranging from trauma to degenerative processes in the context of tendinopathy [2, 3]. The presence of clinical signs such as the Trendelenburg sign and gait instability in patients who have failed conservative treatment has been observed [1]. In such cases, the standard treatment is surgical repair of the gluteal tendon lesions [4, 5]. A number of different surgical techniques for repairing the gluteal tendon have been described [3, 6]. These include endoscopic and open surgical repairs, in addition to double-row and single-row techniques with suture anchors and transosseous repairs [6]. Repair sutures utilising the double-row technique (DR) are gaining importance for gluteal tendon lesions. Research on the suturing technique has indicated several advantages of the DR method. Firstly, it has been observed to distribute pressure more uniformly across the bone compared with the single-row (SR) method. This potential enhancement in tendon healing is a significant consideration in surgical outcomes [7]. Secondly, DR fixation to the bone has been demonstrated to achieve a more stable tendon fixation with higher strength when compared with the SR technique [7]. In conclusion, a survey of extant literature pertaining to the repair of gluteal tendons in the context of DR fixation reveals the existence of a reliable methodology, which has yielded commendable clinical outcomes [6].

However, re-tear failures remain a significant complication in treatment, with a re-tear rate documented in reviews as ranging from 3.8% to 7.4% [6, 8]. The risk of failure is increased in cases of larger tendon tears, retraction of the tendon (> 2 cm), and the occurrence of Trendelenburg gait [9]. This may be due to reduced healing potential or insufficient tendon repair [9].

The biomechanical load requirements on a gluteal tendon suture are of particular interest [7, 10–12]. Achieving a high primary stability of the tendon suture is necessary to avoid failure of the repair suture, particularly in the early rehabilitation phase, and to counteract progressive fatty atrophy through early muscle activation [3, 13, 14].

The aim of this study was to biomechanically compare the stability of two different suturing techniques in an ovine model. The hypothesis is that augmentation in combination with a transosseous-equivalent technique repair (DR +) achieves a significant improvement in stability compared with the conventional DR technique, expressed as an increased ultimate load at failure.

## Material and methods

The decision to utilise the ovine model in this study was predicated on the premise that the tendon length of the gluteus medius muscle (sheep: 51.4 ± 3.9 mm; human: ~50 mm) is anatomically comparable and morphologically similar [15]. In the domain of shoulder surgery and research in rotator cuff pathologies, the ovine model is an established one, despite the presence of some significant anatomical differences [16].

### Specimen preparation

For the present study, pelvic halves (including the proximal femur and surrounding soft tissue) from euthanized adult (> 4 years) female black-headed mutton sheep were utilised. The average body weight was 80.44 ± 8.35 kg (DR) and 76.81 ± 9.76 kg (DR +), respectively. In addition, only female animals of the same breed (blackhead) were used, with no indications of health impairments. Further relevant characteristics have been included in the supplementary material. The sheep were included in experimental studies, which were approved by the Lower Saxony State Office for Consumer Protection and Food Safety, LAVES (approval numbers 33.12-42,502-04-19/3255 and 20/2531).

The inclusion criteria were integrity of the gluteus medius and minimus muscles, their tendons (absence of macroscopic tears or degenerative changes) and integrity of the proximal femur without evidence of fractures or anatomical deformities.

**Fig. 1:**
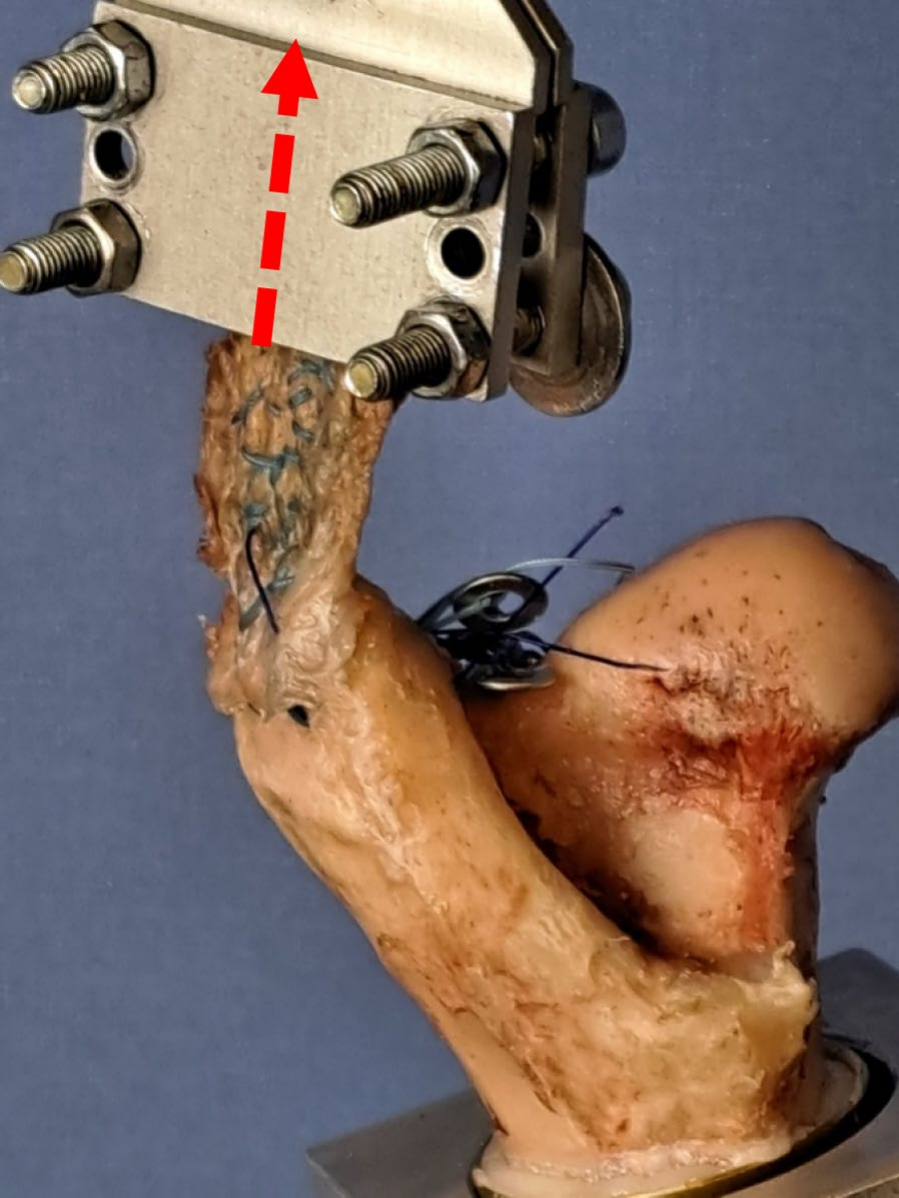
Fixation of the tendon with the tendon-clamp and visualisation of the direction of traction (red arrow) in an ovine model

The specimens were stored at –20 °C and subsequently thawed for 24 h at room temperature prior to preparation and testing. The abductor muscles, gluteal tendons and bones were meticulously prepared with a scalpel and raspatory, and checked for intactness. If the inclusion criteria were met, the gluteus medius muscle was removed from the bony pelvis (ala ossis ilii). The tendon insertion was then carefully separated from the greater trochanter at the footprint using a scalpel to create a standardised full-thickness tendon lesion. Subsequently, the femur was detached from the acetabulum, and the remaining soft tissue was completely removed. Four marks (each 0.5 cm apart) were then placed on the lateral greater trochanter to standardise drill hole positioning. In view of the fact that failure of tendon sutures typically occurs in the tendon area and not in the bony anchorage, commercially available bone anchors were replaced by metal eyebolts at the anterior part of the femoral greater trochanter for anchoring the repair sutures in the bone (Fig. 1).

Muscular tissue was carefully removed from the tendon using a raspatory to ensure sufficient tendon length for secure clamping. The tissue of the specimens was kept moist with physiological saline solution (NaCl 0.9%) throughout the experiment.

### Surgical procedure

The surgical technical implementation of both refixation techniques was carried out by a single experienced orthopaedic surgeon, who specialises in open gluteus tendon repair using the double-row technique. The tendon sutures were performed with a polyester suture material (FiberWire^®^ #2, Arthrex Inc., Naples, FL, USA) in the respective repair technique (DR and DR +) consisting of two rows of suture anchors in the following steps:

Firstly, anatomical tendon position was marked on the footprint, then the knotting position was marked on the tendon, taking the footprint into account.

#### Double row transosseous-equivalent technique (DR)

**Fig. 2:**
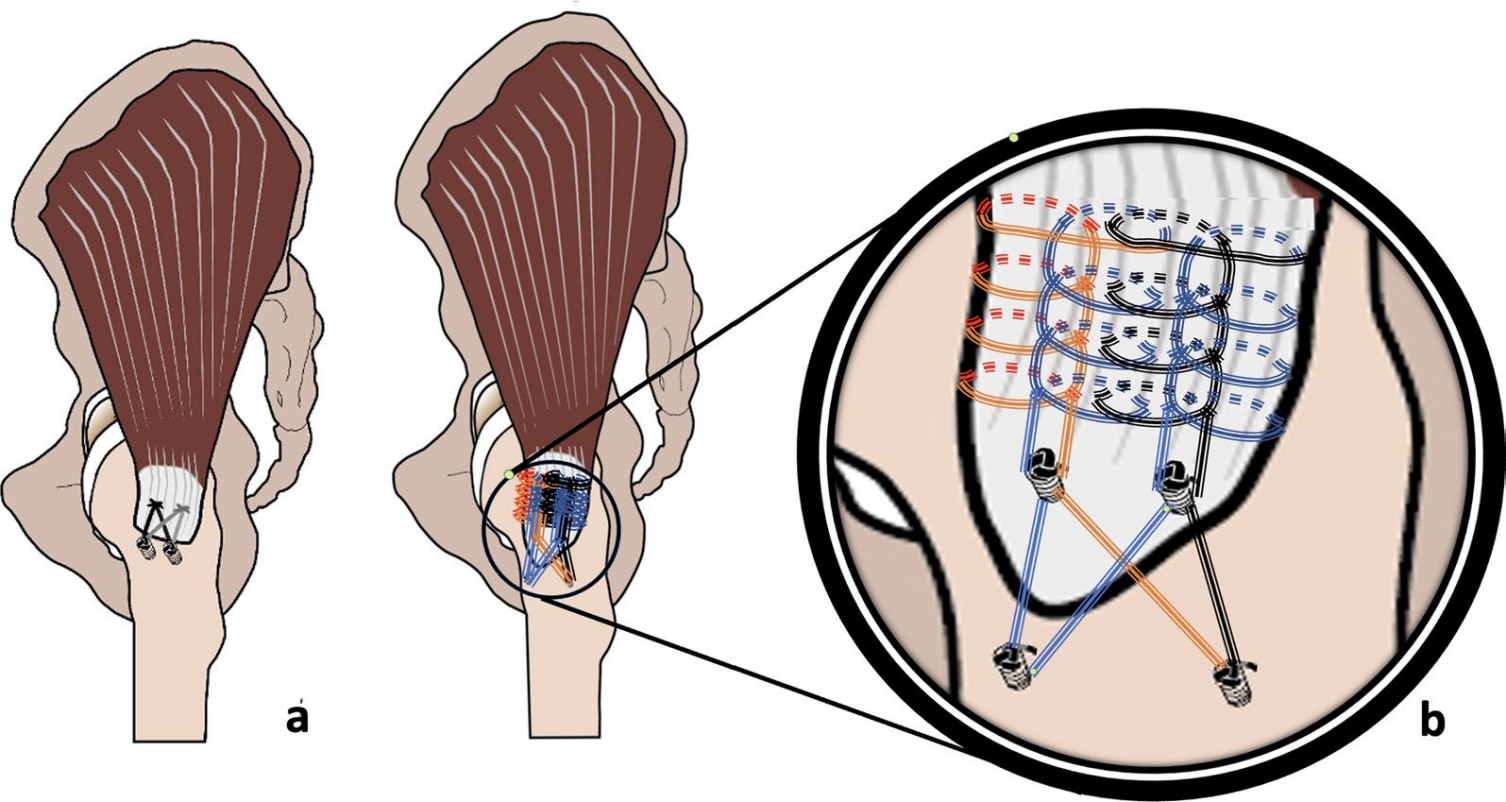
Illustration of the DR (**a**) and DR + (**b**) technique on a human model; in the current study, the depicted anchors were replaced by bony anchoring with metal eyebolts

The sutures were performed in accordance with Park et al. in nine sheep specimens [17]. Following the transosseous-equivalent technique, two sutures were secured to a metal ring at the proximal row, passed through the bone drill holes, and through the tendon at the marked points. They were then crossed in a mattress suture configuration, tensioned towards the distal tendon markings, and passed through the tendon (Fig. 2a). Finally, after threading the sutures through the distal bone holes, they were tied to metal eyebolts.

#### Double-row transosseous-equivalent technique with additional augmentation (DR +)

The DR + technique is equivalent to the DR technique, but supplemented by additional suturing of the proximal tendon area. First, the tendon was sutured over a distance of approximately 4 cm in four rows from proximal to distal, like crossing fir trees (Fig. 2b). The sutures also grasped tendon fibres that were also grasped by the suture in the adjacent row. This resulted in tendon strands which were tensioned by two threads in opposite directions. The sutures were refixed after the proximal area of the tendon and were knotted at the footprint using the described DR technique.

To our knowledge, this augmented technique (DR +) represents a novel method that has not previously been described or biomechanically tested in this specific context.

Following the finalisation of the sutures, the femora were embedded with Rencast FC 52/53 Isocyanate, Polyol FC 53, Filler DT 982 (Huntsman Corp., TheWoodlands, TX, USA) in brass sleeves (diameter 38 mm, length 52 mm, wall thickness 1 mm) and rigidly attached to the testing machine by use of a screw clamp.

### Biomechanical loading tests

All tests were performed on the uniaxial testing machine (Zwick 1445, Zwick-Roell, Ulm, Germany) at room temperature.

The prepared tendons were positioned on a screw clamp so that the testing machine performed the loading tests in the physiologically correct tensile direction (Fig. 1) without changing the position of the femora, and thus, the tensile direction. A custom-made tendon clamp, featuring roughed surface areas, was used to secure the proximal end of the tendon with both sides of the clamp interlocking and tightened with four screws. The sutured area of the tendon was always located outside the clamp. The proximal parts of the tendon that were outside the clamp were cut off with a scalpel close to the clamp to prevent distortion of the results due to slippage.

Initially, the specimens were loaded with a preload of 5 N for 5 s (speed 50 mm/min). This was followed by cyclic preconditioning with 10 N over 10 cycles at a speed of 10 mm/min. The pull-out test was then carried out at a speed of 10 mm/min until failure, while the associated force and displacement were measured. The tests were terminated when the repaired construct failed, resulting in a sudden decrease in force [18].

### Data and statistical analysis

The force and displacement for each specimen were recorded, and the force at failure (N) and stiffness (N/mm) were determined. The force at failure is defined as the maximum force measured in Newtons (N) at the time of failure of the tendon suture. The stiffness (N/mm) was given in the linear range of the load–displacement curve and corresponds to the gradient, referring to the ability of the tendon–suture complex to withstand a tensile force before plastic deformation occurs. A faulty test consisted of the tendon slipping out of the tendon clip during the test before the suture failed, resulting in a drop-out.

The statistical analysis of the biomechanical in vitro test data was conducted using the programming language R 4.1.0 (R Foundation for Statistical Computing) with the software RStudio 1.4.1717 (RStudio, Inc., Boston, MA, USA). The differences between the groups were analysed using the Mann–Whitney *U* test, with a significance level set at *p* = 0.05.

The resulting data were presented as mean ± standard deviation, with the diagrams representing boxplots.

## Results

The mean force at failure was 155.9 ± 53.9 N in the DR group and 698.0 ± 80.3 N in the DR + group. The linear stiffness was 12.4 ± 4.8 N/mm in the DR group and 31.3 ± 14.9 N/mm in DR + group.

**Fig. 3:**
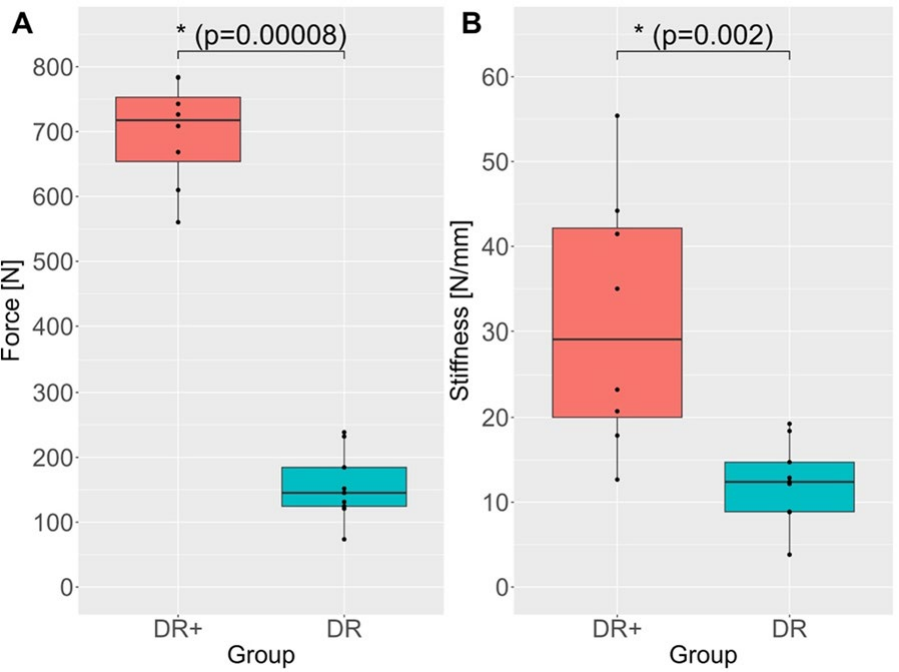
Results of force at failure (**A**) and stiffness (**B**) between the two repair techniques presented as boxplots

The differences between the repair techniques were highly significant for both parameters: force at failure (*p* = 0.00008) and stiffness (*p* = 0.002) (Fig. 3). The primary data from the pull-out tests are provided in the Supplementary Material (Tables S1 and S2), while the statistical data is shown in Table S3.

### Analysing the failure mechanism

#### DR group

In all tests, the tendon slipped out at the side of the tendon’s piercing points, within the suture construct area. In eight cases of the individual tests, the failure occurred at the time of the ultimate load in the area of the proximal rows of sutures of the DR suture. In one case, failure was observed in the distal rows. The sutures and the anchoring in the bone always remained intact. No alterations in the tendon outside the suture area were observed.

#### DR + group

In one case, the tendon slipped out along the tendon fibres in the area of the distal row of sutures. In six cases, a tearing of the tendon was observed just below the fixation clamp in the suture-free area, whereby the knotting and the tendon parts located in this area remained intact. In another case, the sutures of the proximal suture row were torn.

## Discussion

The study examined a new suturing technique with augmentation to extend the gluteal tendon repair in a double-row transosseous-equivalent technique in a full-thickness tear in an ovine model. The results suggest that extending the suture may significantly increase stability by approximately 450% (ultimate load at failure). Furthermore, tensile stiffness exhibited an augmentation of over 250% in favour of the extended technique, thereby supporting the hypothesis that augmentation in combination with a transosseous-equivalent technique repair (DR +) may lead to a significant improvement in stability in an ovine animal model compared with the conventional DR technique.

While the double-row transosseous-equivalent technique has been previously described in detail and is frequently used for gluteal tendon repair, this biomechanical study is the first to use an extended DR technique with additional proximal tendon augmentation [3, 6, 17]. The objective was to achieve the advantages of a DR technique with greater stability. The efficacy of numerous single-row (SR) and DR knot techniques has been reported in the rotator cuff area of the shoulder and, moreover, in the repair of the hip abductors [6, 19]. However, given the significantly higher load requirement and the more challenging follow-up treatment, it is considered important that the procedure provides sufficient stability for which a sufficiently stable suturing technique is essential when compared with the shoulder. For instance, Davies et al. described re-tears of the repair in 24% of cases, as confirmed through magnetic resonance imaging (MRI) [20].

Theoretically, the additional tendon augmentation increases the tension within each suture loop and creates tension between adjacent tendon fibres, potentially improving load distribution. Although similar principles are used in the Krackow suture technique, our augmented method differs by creating continuous interlocking loops that capture multiple adjacent tendon fibres, potentially enhancing overall stability.

The failure mechanism of the isolated DR technique is evident in the proximal (medial) suture row, occurring in eight of the nine specimens. This observation indicates that the main stress occurs in this area due to the parallel alignment of the tendon fibres. In contrast, the DR + technique exhibited a different failure mode, with failure observed predominantly near the fixation clamp area, likely due to clamp-induced damage or tendon thinning from preparation.

Zhu et al. also analysed the timing of gluteal tendon repair in an ovine model [15]. The tendon suture was performed either in the acute situation or 6 weeks after rupture. The development of an animal model with chronic tendinopathy was a part of the study. The repair was performed using two modified Mason–Allen sutures through the anterior and posterior drill. A biomechanical examination was conducted 12 weeks after the repair. The authors observed significant histological and biomechanical changes in the results, with the treatment in the acute situation being superior in both aspects. The biomechanical results indicated 15.4 (± 4.7) N per mm^2^ cross-sectional area in the acute repair group and 14.0 (± 5.8) N in the delayed repair group. A direct comparison with the present study is not feasible owing to the results given in mm^2^ cross-sectional area. An analysis of the failure mode is not described.

A review of the literature revealed four publications that addressed the biomechanics of different repair techniques for gluteal tendon ruptures in human cadaver models [7, 10–12]. While comparisons between human and ovine models are limited owing to anatomical differences, particularly the cross-section of the tendons, these studies provide a comprehensive overview of the biomechanical situation.

Flynn et al. analysed the double-row repair technique, focusing on the knotting technique procedure, comparing the endoscopic approach with the open surgical technique [10]. The ultimate load achieved was 161.1 (± 72.0) N versus 152.1 (± 68.6) N, which is comparable with the results of the DR group from this study. However, the failure mechanism in their work was always found outside the knots, in the area of the musculature. There are two main differences in the procedure compared with this study. Firstly, the musculature was not dissected, so that the traction was performed on the bony pelvis. In most cases, the failure mechanism (92%) was due to tearing of the muscles at the origin. Secondly, a double-row technique was chosen, in which three knotting points were located medially, from which the sutures ran laterally to one knotting point (three-and-one technique). It is unclear whether an optimal two-dimensional footprint reconstruction can be achieved in this way.

Kahlenberg et al. performed double-row and single-row techniques on 12 human fresh frozen cadaveric models and compared their stability [7]. Krackow knots were used as a knotting technique either in one row or in two rows and were compared with each other. In their work, fixation was performed on the muscle using a clamp produced in-house, and the double-row achieved an ultimate load of 348.0 N versus 188.3 N and a linear stiffness of 50.6 N versus 39.5 N/mm. The single-row Krackow knotting achieved similar results to our DR group, with the predominant failure mode in both groups being suture pull-out by the musculotendinous unit, similar to the observation in our DR group. However, the authors did not find a statistically significant difference between the two types of suture tying. It can be hypothesised that the Krackow suture in two rows may exhibit greater stability in comparison with the mattress suture in the medial row used in our study, employing the transosseous-equivalent technique to the lateral row of sutures. To the best of our knowledge, no biomechanical comparative study between the Krackow knotting and DR in transosseous-equivalent technique has confirmed this.

Twardy et al. tested the double-row technique (called the Hip Bridge technique) against the classic Mason–Allen technique in single-row and observed a significantly higher stability in favour of the double-row technique (ultimate load: 339.1 ± 144.4 N versus 209.6 ± 62.1 N) with an elastic deformation of 4.1 ± 1.7 mm versus 5.3 ± 0.5 mm (without significance) [12]. Failure in the double-row group (*n* = 5) occurred exclusively in the tendon area, while in the group using the Mason–Allen technique different forms of failure occurred (tendon failure (1/6), bone cutting (4/6) and muscle rupture (1/6). This is consistent with the failure mechanism observed in our DR technique in the ovine model.

In a study by Dishkin-Paset et al., two distinct double-row techniques were examined (employing massive cuff stitches versus knotless lateral anchors), yielding an ultimate load of 439 N compared with 454 N without statistical significance [11]. However, a specific failure assessment pertaining to the method is not provided, hindering a direct comparison with the generally/apparently high results of the aforementioned publications and our own.

Other factors that influence the stability of a suture construct include trochanteric decortication versus nondecorticated in the suture anchor area and bone mineral density [21]. The rationale behind decortication in the footprint area, where anchors are used to secure the tendon to the bone, is to enhance the healing potential of the repaired tendon to the bone [21]. In their study, Putnam et al. investigated these aspects in 19 human cadavers. Their findings revealed that bone decortication (by 2 mm) of the trochanter in the area of the suture anchors (load to failure in nondecorticated 206.7 ± 75.0 N and in decorticated ± 152.3 ± 60.2 N) and reduced bone mineral density resulted in a significant decrease in the stability of the anchors [21]. In the present study, the focus was on the stability between the tendon and the repair sutures, and suture anchors were not utilised in the experimental setup. The work of Putnam et al. signifies an essential component in the pursuit of optimising the primary stability of the suture construct. Concomitantly, the question of whether the decortication surface is variable, such that the anchors can be positioned in the nondecorticated area without compromising healing potential, remains to be addressed.

On the basis of the data in this study, augmentation, as tested in the ovine model, appears to exceed the results reported in human cadaveric models in terms of ultimate load (698.0 ± 80.3 N versus 152.1–454 N). Given that the footprint area of the sheep is smaller than that of humans in terms of insertion length (15.4 mm versus 43.8 mm) and width (4.6 mm versus 11.7 mm), and human tendon volume is higher [15], it can be hypothesised that the relative differences in human specimens result in even greater strength due to the augmentation itself. However, this hypothesis requires verification in further studies.

A possible disadvantage of the suture technique presented is the need for open surgery. The extended knotting (augmentation) requires more time compared with DR. Furthermore, the extent to which the suture affects tendon nutrition remains to be clarified. Studies of the DR transosseous equivalent technique in the rotator cuff area of the shoulder have shown that even the DR technique leads to reduced but preserved blood flow in the tendon repair site in the early phase [22]. The extent to which additional augmentation further reduces blood supply is still unclear, especially as this suture might partially involve the myotendinous junction of the gluteus medius. Flack et al. showed that the human gluteus medius muscle has significant dimensional variability in humans (length: 112.9–171.0 mm and width: 131.6–158.0 mm) [23]. The fan-shaped muscle can be divided into four compartments on the basis of innervation and structure, with the anterior compartment having the largest volume [23]. These anatomical characteristics explain the wide tendon insertion into the muscle, particularly on the medial side. On the lateral side, the tendon is only superficial just prior to insertion and occurs anterior to the muscle mass. Robertson et al. describe that the insertion of the tendon at the greater trochanter can be divided into two major areas [24]. The lateral facet with an area of 438.0 mm^2^ is approximately twice as large as the superoposterior facet with 196.5 mm^2^. Taking these facts into account, in some cases, muscle parts could be included in the suture technique shown. However, the anatomy of this junction is flat and is therefore not fully utilised. Another aspect is that the tendon often has to be punctured along its course, causing damage that cannot be ignored.

This study has some limitations. Firstly, the study on the ovine model is not fully comparable to humans, as the anatomy is not completely similar [15]. The tendons in the animal model in this study were not degenerated, although this is much more common clinically [25]. Degenerative changes in tendinopathy lead to structural changes in the tendon that reduce the load bearing capacity and can lead to damage at low elongation [15, 25]. Furthermore, the focus of this study was on the stability of the knotting between the tendon and the repair suture, and therefore the stability of the bone anchorage was not investigated, which is also an important factor in the stability of tendon repair [21]. In addition, the small size of the sample has to be mentioned as a limitation of our study. Although there are some uncertainties that need to be verified in the future, this study provides an important answer to the question of primary stability in complete tendon lesions using combined tendon augmentation during repair.

With regard to applicability in humans, factors such as anatomical or functional differences may need to be considered. Although this biomechanical ovine model provides important insights in this research field, there are differences in tendon dimensions and the biomechanical function of the musculature that need to be considered. Nevertheless, the significant improvement in the mechanical stability of DR + can be assumed to have potential benefits, particularly during early postoperative rehabilitation, which enables more intensive physiotherapeutic interventions that counteract potential muscle atrophy and reduce the risk of tendon re-rupture, especially in this postoperative phase. However, these aspects require further clinical studies for applicability in humans.

## Conclusions

In the ovine model, the double-row technique combined with additional proximal tendon augmentation significantly improved stability compared with the conventional double-row technique. This effect can be attributed to the increased tension generated by counteraction of the sutures and tendon bundles, coupled with the increasing force development of the muscle. As a result, slippage of tendon fibres from the threads is reduced in DR + compared with the DR suture. The general advantages of the double-row transosseous-equivalent technique are maintained, which allows a widespread pressing of the tendon stump against the footprint.

## Supplementary Information

Additional file 1.

## Data Availability

The datasets used and analysed during the current study are available from the corresponding author on reasonable request.

## Abbreviations

SR: Single-row suture technique
DR: Double-row suture technique
DR +: Double-row suture technique with additional augmentation
