# Supplementary material for: A novel augmentation technique for the repair of full thickness gluteal tendon tears: a biomechanical analysis in an ovine model
Source: J Orthop Traumatol. 2025 May 24;26:33. doi: 10.1186/s10195-025-00850-1 (PMC12103436; doi:10.1186/s10195-025-00850-1)
Supplement: Supplementary file 1 — Additional file 1. [file 10195_2025_850_MOESM1_ESM.docx]

**Supplementary material**

**Table S1: Primary data of specimens ( DR-group)**

| # | ID | age [years] | weight [kg] | side | race | sex | approval number | **Force [N]** | **Stiffness [N/mm]** |
| --- | --- | --- | --- | --- | --- | --- | --- | --- | --- |
| DR1 | ID-1519 | > 4 | 87.5 | right | blackhead | female | 19/3255 | 184.56 | 8.83 |
| DR2 | ID-1523 | > 4 | 82.0 | right | blackhead | female | 19/3255 | 120.96 | 3.82 |
| DR3 | ID-1520 | > 4 | 87.5 | left | blackhead | female | 19/3255 | 232.16 | 19.25 |
| DR4 | ID-1549 | > 4 | 79.5 | left | blackhead | female | 19/3255 | 124.48 | 12.88 |
| DR5 | ID-1544 | > 4 | 66.5 | left | blackhead | female | 19/3255 | 238.64 | 12.40 |
| DR6 | ID-1554 | > 4 | 86.0 | left | blackhead | female | 19/3255 | 73.60 | 14.72 |
| DR7 | ID-1565 | > 4 | 82.5 | right | blackhead | female | 20/3531 | 145.28 | 12.17 |
| DR8 | ID-1545 | > 4 | 66.5 | right | blackhead | female | 19/3255 | 151.84 | 18.40 |
| DR9 | ID-1557 | > 4 | 86.0 | right | blackhead |  | 19/3255 | 131.28 | 8.87 |
|  |  | >4 | 80.44 ± 8.35 | r:5, l:4 |  |  |  | 155.87 ± 53.85 | 12.37 ± 4.84 |

**Table S2: Primary data of specimens (DR+ -group)**

| # | ID | age [years] | weight [kg] | side | race | sex | approval number | **Force [N]** | **Stiffness [N/mm]** |
| --- | --- | --- | --- | --- | --- | --- | --- | --- | --- |
| DR+1 | ID-1562 | > 4 | 84.0 | left | blackhead | female | 20/3531 | 668.48 | 17.86 |
| DR+2 | ID-1512 | 6 | 70.5 | right | blackhead | female | 19/3255 | 560.32 | 12.67 |
| DR+3 | ID-1515 | 6 | 58.0 | left | blackhead | female | 19/3255 | 742.88 | 44.20 |
| DR+4 | ID-1568 | > 4 | 82.0 | left | blackhead | female | 20/3531 | 783.20 | 23.27 |
| DR+5 | ID-1570 | > 4 | 78.0 | left | blackhead | female | 20/3531 | 609.92 | 20.72 |
| DR+6 | ID-1538 | > 4 | 79.0 | left | blackhead | female | 20/3531 | 726.56 | 55.41 |
| DR+7 | ID-1561 | > 4 | 90.0 | right | blackhead | female | 19/3255 | 708.64 | 35.01 |
| DR+8 | ID-1518 | > 4 | 73.0 | left | blackhead | female | 19/3255 | 784.00 | 41.46 |
|  |  |  | 76.81 ± 9.76 | r:2, l:6 |  |  |  | 698 ± 80.34 | 31.33 ± 14.97 |

**Table S3: Statistical data of both groups**

|  | **Force [N]** | | **Stiffness [N/mm]** | |
| --- | --- | --- | --- | --- |
|  | DR+ | DR | DR+ | DR |
| n | 8 | 9 | 8 | 9 |
| mean | 698.00 | 155.87 | 31.33 | 12.37 |
| SD | 80.34 | 53.85 | 14.97 | 4.84 |
| median | 717.60 | 145.28 | 29.14 | 12.40 |
| min | 560.32 | 73.60 | 12.67 | 3.82 |
| max | 784.00 | 238.64 | 55.41 | 19.25 |
| range | 223.68 | 165.04 | 42.73 | 15.43 |
| 25% quantile | 653.84 | 124.48 | 20.01 | 8.87 |
| 75% quantile | 752.96 | 184.56 | 42.15 | 14.72 |
